# *Prostate-Specific Ets* (*PSE*) factor: a novel marker for detection of metastatic breast cancer in axillary lymph nodes

**DOI:** 10.1038/sj.bjc.6600190

**Published:** 2002-03-18

**Authors:** M Mitas, K Mikhitarian, L Hoover, M A Lockett, L Kelley, A Hill, W E Gillanders, D J Cole

**Affiliations:** Department of Surgery, Suite 420, Medical University of South Carolina, Charleston, SC 29425, USA; Department of Surgery, National University of Ireland, Dublin, Ireland

**Keywords:** virtual Northern blot, receiver operator characteristic curve, gene overexpression, SYBR Green I, real-time RT–PCR

## Abstract

*Prostate Specific Ets* factor is a recently identified transcriptional activator that is overexpressed in prostate cancer. To determine whether this gene is overexpressed in breast cancer, we performed a virtual Northern blot using data available online at the Cancer Genome Anatomy Project website. Ninety-five SAGE libraries were probed with a unique sequence tag to the *Prostate Specific Ets* gene. The results indicate that *Prostate Specific Ets* is expressed in 14 out of 15 breast cancer libraries (93%), nine out of 10 prostate cancer libraries (90%), three out of 40 libraries from other cancers (7.5%), and four out of 30 normal tissue libraries (13%). To determine the possibility that the *Prostate Specific Ets* gene is a novel marker for detection of metastatic breast cancer in axillary lymph nodes, quantitative real-time RT–PCR analyses were performed. The mean level of *Prostate Specific Ets* expression in lymph nodes containing metastatic breast cancer (*n*=22) was 410-fold higher than in normal lymph node (*n*=51). A receiver operator characteristic curve analysis indicated that *Prostate Specific Ets* was overexpressed in 18 out of 22 lymph nodes containing metastatic breast cancer (82%). The receiver operator characteristic curve analysis also indicated that the diagnostic accuracy of the *Prostate Specific Ets* gene for detection of metastatic breast cancer in axillary lymph nodes was 0.949. These results provide evidence that *Prostate Specific Ets* is a potentially informative novel marker for detection of metastatic breast cancer in axillary lymph nodes, and should be included in any study that involves molecular profiling of breast cancer.

*British Journal of Cancer* (2002) **86**, 899–904. DOI: 10.1038/sj/bjc/6600190 www.bjcancer.com

© 2002 Cancer Research UK

## 

Given the heterogeneity of metastatic breast cancer, it is unlikely that one breast cancer-associated gene will be overexpressed in all disease tissues. We have shown that a multi-marker RT–PCR assay can increase the sensitivity of metastatic detection (Mitas *et al*, 2001). There is also evidence that the use of multiple markers may also increase the specificity of RT–PCR if more than one marker must be positive for a positive test result (Houghton *et al*, 2001; Hu and Chow, 2001). These data provide the impetus to identify novel markers for the detection of metastatic breast cancer.

Testing newly discovered genes that are potential diagnostic markers for breast cancer is a time-consuming process; it requires that gene-specific primers be designed, screened, and then validated on a statistically significant number of control normal and pathology-positive tissues. In theory, the testing process could be expedited if expression data for informative genes were available. Fortunately, modern methods of genomics have recently produced an unprecedented amount of expression data from normal and cancer-associated tissues. As part of the Cancer Genome Anatomy Project (CGAP), the National Center for Biotechnology Information recently created a public website (http://www.ncbi.nlm.nih. gov/ncicgap/) that allows users to compare computed gene expression profiles on-line between selected cDNA libraries, thus generating a Digital Differential Display (DDD). Using DDD, Scheurle *et al* (2000) recently compared breast cancer tissue with others and concluded that *mammaglobin 1* (*mam1*) was present exclusively in breast cancer tissue. This conclusion is consistent with our real-time RT–PCR results in which we discovered that of 12 breast cancer-associated genes, *mam1* had the highest diagnostic accuracy for metastatic disease in lymph node tissue (Mitas *et al*, 2001). Scheurie *et al*, also concluded that with respect to normal breast tissue, two genes that were the most significantly upregulated in breast cancer were *Prostate Specific Ets* factor (*PSE*) and myosin light chain regulatory polypeptide 5 (*MYL5*).

In this study, we assessed the utility of *PSE* and *MYL5* as markers for quantitative real-time RT–PCR studies by first performing an on-line analysis of the SAGE library database available at the CGAP web site. Serial analysis of gene expression, or SAGE, is designed to gain a direct and quantitative measure of gene expression (Yamamoto *et al*, 2001). The SAGE method is based on the isolation of unique sequence tags 9–10 bases in length which are located immediately adjacent to the most 3′ CATG or GATC sequence of the mRNA. The tags are serially concatenated into long DNA molecules and the sequence information derived from them is deposited as a library into the SAGE database maintained by the CGAP. Each of the 95 functional libraries in the existing database can be compared to one another, or alternatively, can be queried using unique sequence tags. The output from such a query is obtained immediately, and the data is expressed and normalised as number of sequence tags per million library bases. This process represents a virtual Northern blot (VNB) of various normal and cancer-associated tissues.

We first present VNB data that *PSE*, but not *MYL5*, is highly overexpressed in breast cancer tissue. Using real-time RT–PCR, we further demonstrate that *PSE* is highly expressed in four out of five breast cancer cell lines. To determine the potential clinical relevance of the *PSE* gene as a molecular diagnostic marker, we analysed a panel of normal control lymph nodes (*n*=51), and axillary lymph nodes (*n*=22) containing metastatic breast cancer. A receiver operator characteristic curve analysis indicates that the *PSE* gene is capable of detecting metastatic breast cancer in axillary lymph nodes with high accuracy. This analysis also indicates that *PSE* is overexpressed in 18 out of 22 lymph nodes containing metastatic breast cancer, and in three out of 22 pathology-negative lymph nodes from breast cancer patients. These results provide strong evidence that *PSE* is a potentially valuable marker for detection of metastatic breast cancer in axillary lymph nodes and should be integrated as a component of any multi-marker diagnostic assay.

## MATERIALS AND METHODS

### Virtual Northern blot

The cDNA sequences for the *PSE* and *MYL5* genes (GenBank accession numbers XM_004328 and NM_002477, respectively) were used to identify sequence tags (UniGene cluster Hs.79414 for *PSE* and Hs.170482 for *MYL5*) at the NCBI Virtual Northern blot website (www.ncbi.nlm.nih.gov/SAGE/sagevn.cgi). The sequence tags were then used to determine levels of expression in the 95 functional libraries in the SAGE database at the CGAP. *Nla*III was used as the anchoring enzyme.

### Tissue specimens

Tissue specimens were obtained in accordance with federal and institutional guidelines as previously described (Mitas *et al*, 2001).

#### Axillary lymph nodes from breast cancer patients

Axillary lymph nodes were obtained from breast cancer patients undergoing surgical therapy for the treatment of breast cancer. Axillary lymph nodes were evaluated immediately by a surgical pathologist at the time of specimen acquisition. For the purposes of RT–PCR analysis, the lymph nodes were identified and bisected. One portion was processed for real-time RT–PCR analysis, and the other portion was sent for routine pathologic analysis. Lymph node specimens destined for RT–PCR analysis were immediately snap frozen in liquid nitrogen by the Hollings Cancer Center (HCC) Tissue Procurement/Tissue Bank technician to prevent RNA degradation. These nodes were stored at −70°C in the HCC Tissue Bank until total RNA processing could be performed.

#### Lymph nodes from patients without evidence of cancer

Lymph nodes were obtained from 51 patients undergoing elective carotid endarterectomy. None of the patients had any history or clinical evidence of cancer. At the time of the procedure, a single cervical lymph node was removed and processed as noted above.

#### Normal breast tissue

Normal breast tissue specimens were obtained from patients at St Vincent's University Hospital (Ireland) undergoing breast surgery for benign and malignant breast disease. All tissue specimens were evaluated immediately at time of excision by a surgical pathologist and normal breast tissue was identified. In cases where the procedure was performed for malignant disease, normal breast tissue was obtained from a part of the specimen at least 3 cm from the malignant disease. Tissues were then snap frozen in liquid nitrogen, stored at −70°C. Specimens were shipped overnight to MUSC in dry ice.

### RNA isolation

Total cellular RNA was isolated using a guanidium thiocynate-phenol-chloroform solution as previously described (Mitas *et al*, 2001). Briefly, a single tissue specimen was removed from −70°C storage and weighed as quickly as possible without allowing the tissue to thaw. Tissue (0.15 gm) was then homogenised in 1.5 ml of a guanidium thiocynate-phenol-chloroform solution (Tel-Test, Friendswood, TX, USA) using a model 395 type 5 polytron (Dremel, Racine, WI, USA). Total RNA was then isolated as per the manufacturer's instructions. Final RNA pellets were dissolved in 50 μl of DEPC treated water. RNA yield was determined by spectroscopy. Complementary DNA was made from 5 μg of total RNA using M-MLV reverse transcriptase (Promega, Madison, WI, USA).

### Determination of* PSE* intron/exon boundaries

To define the intron/exon boundaries of the *PSE* gene, its genomic sequence (GenBank accession number AL157372) was aligned with its cDNA sequence (GenBank accession number XM_004328) using Blast 2 software (www.ncbi.nlm.nih.gov/BLAST/).

### Real-time RT–PCR

Real-time RT–PCR was performed on a Gene Amp 5700 Sequence Detection System (PE Biosystems, Foster City, CA, USA) as previously described (Mitas *et al*, 2001). Briefly, standard reaction volume was 10 μl and contained 1× SYBR RT–PCR buffer, 3 mM MgCl_2_, 0.2 mM each of dATP, dCTP, dGTP, 0.4 mM dUTP, 0.005 U AmpliTaq Gold, 0.002 U AmpErase UNG erase enzyme, 0.35 μl cDNA template, and 50–900 nM of oligonucleotide primer. Initial steps of RT–PCR were 2 min at 50°C for UNG erase activation, followed by a 10-min hold at 95°C. Cycles (*n*=40) consisted of a 15 s melt at 95°C, followed by a 1 min annealing/extension at 60°C. The final step was a 60°C incubation for 1 min. All reactions were performed in triplicate. Threshold for cycle of threshold (C_t_) analysis of all samples was set at 0.5 relative fluorescence units. The following primer pair for the *PSE* gene was used: forward, AGTGCTCAAGGACATCGAGACG and reverse, AGCCACTTCTGCACATTGCTG.

For a given real-time RT–PCR sample, the point at which fluorescence starts to increase above background is referred to as the cycle threshold (C_t_) value. The C_t_ value is therefore inversely proportional to the amount of specific mRNA species in the original tissue sample. In our analyses, we normalised the results to a reference control gene, β_*2*_-*microglobin*, to control for RNA preparation. The result is termed the ΔC_t_ value, in this case the difference in C_t_ values for the *PSE* gene and the β_*2*_-*microglobin* control gene. High ΔC_t_ values are correlated with low levels of gene expression whereas low ΔC_t_ values are correlated with high levels of gene expression. Relative *PSE* gene expression levels in two samples can be derived from real-time RT–PCR data using the equation (1+AE)^ΔΔCt^ (PE Biosystems, 1998), where AE is the amplification efficiency of the *PSE* gene, and ΔΔC_t_ is the difference between ΔC_t_ values from two cell/tissue samples. To determine the AE of the *PSE* amplicon, real-time RT–PCR studies were performed using serial 10-fold dilutions of LNCaP cDNA using the formula AE=10^1/m^-1 (Bieche *et al*, 1999), where m is the slope determined by linear regression analysis.

### Receiver operator characteristic (ROC) curve analysis of cancer associated genes

Matched (control normal and metastatic breast cancer) data sets were analysed using ROCKIT 0.9B Beta Version software (Metz *et al*, 1998). Sensitivity and 1-Specificity values obtained from ROCKIT 0.9B software files were then imported into Microsoft Excel for further analysis.

## RESULTS

### Virtual Northern blot of *PSE* gene

To investigate whether the *PSE* or *MYL5* genes were overexpressed in breast cancer, we performed a virtual Northern blot (VNB) using an electronic database available at the CGAP website (www.ncbi.nlm.nih.gov/SAGE/index.cgi). The cDNA sequences for the *PSE* and *MYL5* genes were used to identify reliable sequence tags to the respective UniGene clusters Hs.79414 and Hs.170482. The VNB revealed that two or more tags to the *MYL5* gene were present in only three prostate, one kidney, one brain, but no breast (or other) libraries (data not shown). In contrast, two or more tags to the *PSE* gene were identified in 14 out of 15 breast cancer libraries (93%), nine out of 10 prostate cancer libraries (90%), but only three out of 40 libraries from other cancers (7.5%), and four out of 30 normal tissue libraries (13%) (Table 1

**Table 1:**
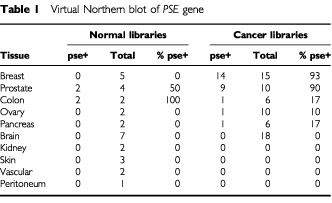
Virtual Northern blot of *PSE* gene

). *PSE* was detected in two out of two normal colon libraries (100%), and two out of four normal prostate libraries (50%). No expression of the *PSE* gene was observed in five normal breast tissue libraries. The observation that *PSE* was expressed in colon tissue agrees well with previous Northern blot results of Yamada *et al* (2000) who showed that *PSE* was significantly expressed in mouse prostate and cecum, and to a minor extent in the oviduct, seminal vesicle, stomach, and colon. The tissue expressing the highest level of *PSE* (664 *PSE* tags per million (TPM) sequenced tags) was a lymph node containing metastatic breast cancer (data not shown). For comparison, this same tissue expressed β_*2*_-*microglobin* at a rate of 3320 TPM (data not shown). This highly expressed gene is often used as an internal reference control for gene quantification. The observation that the rate of tag detection for *PSE* and β_*2*_-*microglobin* genes differed only five-fold provides evidence that *PSE* is highly overexpressed in at least some breast cancer tissues. The results of the electronic screening analysis provided the impetus to formally test *PSE* as a potential molecular marker of metastatic breast cancer.

### Identification of a PCR primer pair specific for the *PSE* gene

**Figure 1:**
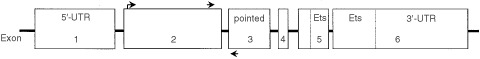
Genomic structure of the *PSE* gene. Intron/exon boundaries of the *PSE* gene were obtained as described in Materials and Methods. The nucleotide lengths of exons 1–6 are: 1 (386), 2 (465), 3 (198), 4 (48), 5 (146), 6 (650). Lengths (in nucleotides) of introns following respective exons are: 1 (11 444), 2 (2338), 3 (1421), 4 (118), and 5 (797). Functional domains of the exons (Oettgen *et al*, 2000) are indicated in the top portion of the boxes. Translation start site is indicated by arrow at far left of exon 2. Arrow at the top right of exon 2 indicates approximate position of forward primer used for real-time RT–PCR analyses is indicated, while arrow below exon 3 indicates position of the reverse primer.

We designed four intron-spanning primers to the *PSE* gene using Primer Express software. To validate the primers, we performed real-time RT–PCR analyses using cDNA prepared from LNCaP, a prostate cell line which has previously been shown to express the *PSE* gene at high levels (Nozawa *et al*, 2000). Real-time RT–PCR analyses were performed with SYBR Green I, a fluorescent dye that binds to double-stranded DNA (Schneeberger *et al*, 1995). With each of the four primer pairs, we observed that *PSE* expression levels in LNCaP cells were comparable to the control housekeeping gene, β_*2*_-*microglobin* (data not shown; in a typical cell, β_*2*_-*microglobin* comprises 0.1–1% of the total mRNA). To avoid potential amplification of genomic DNA in subsequent studies, we selected the primer pair that spanned the largest intron (2.3 Kb; Figure 1

). Using this primer pair and LNCaP cDNA, we confirmed amplification of a specific product by obtaining the following results: (1) on-line fluorescence detection of amplification following cDNA preparation in the presence but not the absence of reverse transcriptase, (2) the measured melting temperature (T_m_) of the product (as determined by denaturation profile analysis) was 84°C, a value within 2°C of its calculated T_m_ (83°C), and (3) the sequence of the amplified fragment matched the *PSE* cDNA gene sequence (data not shown). For all subsequent real-time RT–PCR studies described in the text, specific amplification of the *PSE* cDNA was verified by denaturation profile analysis.

### *PSE* is highly expressed in various breast cancer cell lines

**Figure 2:**
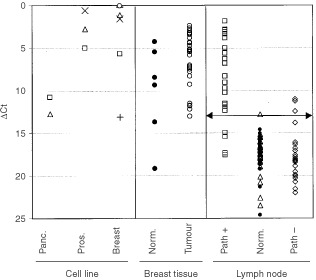
Expression of *PSE* in cancer cell lines, breast tissue, and lymph nodes. Real-time PCR analyses were performed using primers to the *PSE* gene and various cell line or tissue samples described in Materials and Methods. C_t_ values for each gene were determined from triplicate reactions. ΔC_t_ values were obtained by subtracting the mean C_t_ value of β_*2*_- *microglobin* for the respective sample from the mean C_t_ value for *PSE*. Symbols for the cell lines are: Pancreas: triangle, Panc1; open square, Capan1. Prostate: open square, DUI145; X, LnCap; triangle, PC3. Breast: open circle, MDA453; open square, MDA231; −, MDA361; triangle, SkBr3; X, MCF7; +, MCF10A. Number of samples analysed for each tissue: 51 negative control normal lymph nodes (solid circles=females, open triangles=males), 22 pathology-positive axillary lymph nodes (open square) and 22 pathology-negative axillary lymph nodes (open diamond). Horizontal line indicates ΔC_t_ threshold value obtained at a 98% specificity level using the ROC curve analysis described in the text.

Relative expression of the *PSE* gene was determined in six breast, two pancreatic, and three prostate cancer cell lines by real-time RT–PCR analyses. Of the three prostrate cell lines examined, the highest level of *PSE* expression was observed in LNCaP (ΔC_t_=0.6; i.e., *PSE* was expressed at 2^−0.6^-fold (or 0.66) the level of β_*2*_-*microglobin*), whereas the lowest level was observed in DU145 (ΔC_t_=5.2; Figure 2

). In the two pancreatic cell lines, ΔC_t_ values for the *PSE* gene were relatively high (10.8 and 12.7), indicating that this gene was expressed 990-fold lower, and 3600-fold lower respectively than LNCaP. In two of the breast cancer metastatic cell lines, ΔC_t_ values for the *PSE* gene were lower compared to LNCaP (−0.3, MDA-453 and 0.25, MDA-361), indicating that high expression of *PSE* is not restricted to prostate cancer. This result confirms the VNB data. Of interest, the ΔC_t_ value for *PSE* was relatively high (indicating poor expression) in MCF-10A, an immortalised breast cancer cell line considered to have no metastatic potential (Plopper *et al*, 1998).

### *PSE* expression levels in normal lymph nodes and in lymph nodes containing metastatic breast cancer

To assess the potential of *PSE* as a molecular marker for the detection of metastatic breast cancer, we performed analyses on an extensive panel of control lymph nodes, normal breast tissue samples, primary breast cancer samples, and lymph nodes containing metastatic breast cancer. We first analysed 51 lymph nodes from control patients without evidence of cancer. We observed that ΔC_t_ values ranged from 24.6 to 12.8 (mean=17.6), indicating that *PSE* was expressed poorly or not at all in some patients, and at detectable but low levels in others (Figure 2). In 22 lymph nodes containing breast cancer, the ΔC_t_ values varied from 17.0 to 2.0 (mean=8.9); the mean level of expression in metastatic tissue was 410-fold higher compared to normal lymph nodes. This result confirms the potential of *PSE* as a molecular marker for the detection of metastatic breast cancer. In six normal breast tissue samples, ΔC_t_ values for *PSE* ranged from 19.2 to 4.3 (mean=10.1±5.6), providing evidence that although the level of expression of this gene varied in normal breast tissue samples, it was generally higher than normal lymph nodes (mean level of expression 160-fold higher). In 27 primary breast cancer samples, ΔC_t_ values for *PSE* ranged from 13.0 to 2.4 (mean=6.6±3.2). Thus, the mean expression of *PSE* was increased 11-fold in primary breast tumours compared to unmatched normal breast tissue.

To rigorously define the diagnostic value of the *PSE* gene, we performed a receiver operator characteristic (ROC) curve analysis, the most commonly used method for assessing the accuracy of diagnostic tests (Henderson, 1993). ROC curve analysis is based on a plot of sensitivity as a function of 1-specificity. The area under the ROC curve (W) is a measure of diagnostic accuracy such that values between 0.5 and 0.7 indicate low accuracy, values between 0.7 to 0.9 indicate moderate accuracy and values greater than 0.9 indicate high accuracy (Swets, 1988). With respect to metastatic breast cancer, the diagnostic value of *PSE* was 0.949 (95% confidence interval range=0.828–0.990), indicating that PSE was highly accurate. Using a threshold corresponding to a specificity value of 98%, we observed that 18 out of 22 (82%) of the pathology-positive lymph nodes overexpressed *PSE* (Figure 2).

### Detection of occult micrometastatic disease in breast cancer patients

To determine whether *PSE* might prove useful for the detection of occult micrometastatic disease in breast cancer patients, 22 axillary lymph nodes from breast cancer patients without pathologic evidence of metastatic breast cancer were analysed. Previous analysis has revealed that of the 22 nodes, six overexpressed *PIP*, three overexpressed *mam*, and one overexpressed *CEA*. We observed that in three of the 22 samples (14%), *PSE* was overexpressed (Figure 2). All three samples in which *PSE* was overexpressed also overexpressed either *mam* or *PIP* (data not shown). In the remaining 19 samples, *PSE* was expressed at levels not considered significantly different from those observed in normal lymph nodes.

## DISCUSSION

The goal of breast cancer staging is to classify patients by the extent of disease into groups with similar clinical outcomes. Staging facilitates patient management, allowing clinicians to tailor therapies to individual patients. The presence of metastatic disease in the axillary lymph nodes (ALN) is considered the single most important prognostic indicator for breast cancer patients, with an inverse relationship existing between the number of lymph nodes positive for cancer and prognosis (Valagussa *et al*, 1978). As a result, staging for newly diagnosed clinical Stage I and II breast cancer patients has traditionally included an ipsilateral axillary lymph node dissection. Currently, routine pathologic analysis of axillary lymph nodes in breast cancer patients consists of the preparation of one or two sections from the central area of the node followed by staining with hematoxylin and eosin and microscopic examination. However, this procedure evaluates only a small portion of the node, and studies have shown that serial sectioning and immunohistochemistry increase the sensitivity of detection of metastatic disease. The presence of metastatic disease detected in this fashion has been shown to be associated with decreased disease-free survival (McGuckin *et al*, 1996). Although these techniques provide the ability to detect clinically significant metastatic disease, they are extremely time-consuming and expensive, and are not used on a routine basis. The current limitations of breast cancer staging suggest that the successful development and validation of a sensitive and specific molecular diagnostic assay is likely to have a significant clinical impact.

For negative controls, we used cervical (as opposed to axillary) lymph nodes obtained during elective carotid endarterectomy. Unfortunately, we did not have access to normal axillary lymph nodes, which would have been more appropriate for this study. Nonetheless, we feel that cervical lymph nodes served as a sufficient negative control for the genes described in this study. We base this statement primarily on the distribution of ΔC_t_ values in the control negative and pathology-negative groups. In theory, if a given gene is cancer- or tissue-specific, its distribution pattern should be such that with the exception of a distinct subset of high expressing samples in the pathology-negative group, expression patterns should be otherwise identical between pathology-negative and control normal groups. In support of this hypothesis, we observed that the mean ΔC_t_ value for *PSE* gene in the control normal group was 17.7±2 .4, slightly lower (thus corresponding to higher expression) but not statistically different from the mean value in the pathology negative lymph node group (18.7±2.3). In further support, we observed that the pathology-negative group contained four high expressing samples, whereas only one was observed in the control normal group.

We have focused our attention in this study on *PSE*, a gene known to be overexpressed in prostate cancer (Oettgen *et al*, 2000; Yamada *et al*, 2000). We first performed a VNB of the CGAP SAGE database and obtained compelling evidence that *PSE* is overexpressed in various breast cancer tissues (Table 1). The results of this study provide evidence that the gene expression data contained in electronic databases at the CGAP can be used to identify potentially informative marker genes overexpressed in breast and other cancers. In addition to the VNB analysis, the SAGE database can be used to perform DDD where specific cDNA frequencies in normal tissue and cancer-associated libraries are compared. For example, Argani *et al* (2001) recently used the CGAP SAGE database to identify genes that were differentially expressed between pancreatic cancers and normal tissues. They determined that prostate stem cell antigen was overexpressed in pancreatic cancer and confirmed this finding by subsequent RT–PCR and immunohistochemistry analyses of primary pancreatic cancer specimens.

It is not clear whether *PSE* plays a role in the maintenance or progression of breast cancer. *PSE* is a member of the Ets transcriptional family. Ets proteins contain a conserved amino acid DNA binding domain, function in the nucleus, have a short half-life, and are present at low abundance in the cell (Watson *et al*, 1988; Seth *et al*, 1992; Janknecht and Nordheim, 1993; Wasylyk *et al*, 1993). Despite overexpression of *PSE* mRNA in the prostate cancer cell lines DU145, PC-3, and LNCaP, Nozawa *et al* (2000) failed to detect by Western blot analysis any PSE protein in these cell lines. This suggests that in prostate cancer, expression of the PSE gene product is (i) controlled at the translational level and (ii) not necessary for metastatic growth. We suspect that in breast cancer, PSE protein is regulated in a similar manner and that overexpression of *PSE* mRNA likely occurs as a consequence of progression to a metastatic state but is not a causative factor. If this proves to be the case, the gene product is not likely to be a clinically useful therapeutic target.

One of the strengths of molecular diagnostic assays for the detection of breast cancer is the ability to incorporate multiple genetic markers into a single assay. We have already identified five markers with moderate to high accuracy for the detection of breast cancer as defined by ROC analysis (Mitas *et al*, 2001). The use of multiple markers has the potential to increase both the sensitivity and specificity of these molecular diagnostic assays. The results presented here confirm this concept. We have previously analysed 17 of the 22 lymph nodes containing metastatic breast cancer described here with a seven marker panel and have observed that with the exception of one node, all others overexpressed at least two cancer-associated genes (Mitas *et al*, 2001). The remaining node marginally overexpressed *mam1* (28-fold higher than normal control lymph nodes). In this report, we observed that the same node overexpressed *PSE* at a level 1.5×10^5^-fold higher than normal control lymph nodes (data not shown). These results suggest that *PSE* has the capacity to complement the markers that we have previously identified.

We believe that real-time RT–PCR will significantly improve molecular diagnostic assays for the detection of breast cancer. Traditional RT–PCR techniques are at best semi-quantitative, and it has been difficult to differentiate between baseline levels of gene expression in normal tissues and increased levels of gene expression associated with breast cancer, raising the concern for false positive results. Real-time RT–PCR allows for the sensitive detection and quantitation of gene expression and is quickly becoming recognised as the technology of choice for the precise measurement of gene expression levels (Bieche *et al*, 1998; Mitas *et al*, 2001). The amplification conditions for real-time RT–PCR are also standardised, making this an ideal platform for high throughput analysis of multiple markers. The standardised nature of real-time RT–PCR also makes it possible to meaningfully compare the diagnostic accuracy of different markers. In this study, we analysed an extensive panel of control normal lymph nodes and lymph nodes containing metastatic breast cancer. We determined from ROC curve analysis that the diagnostic accuracy of the *PSE* gene was 0.949, a value higher than six of the seven genes previously identified as the best diagnostic markers for the detection of metastatic breast cancer ALNs (Mitas *et al*, 2001). These results provide strong evidence that *PSE* is a potentially informative novel marker, and should be a component of any multiple marker diagnostic assay for detection of breast cancer.
